# GPI: A pan-cancer biomarker integrating genetic alterations, immune infiltration, and therapeutic response prediction

**DOI:** 10.1016/j.gmg.2026.100127

**Published:** 2026-09-10

**Authors:** Wei Hu, Yu Gao, Zimu Li, Zelei Du, Jincheng Tao, Anastasios A. Politis, Fulvio Vincenzo Grilli, Alessandro Di Stefano, Xiefeng Wang, Junxia Zhang, Jianxing Yin

**Affiliations:** a Department of Neurosurgery, The First Affliated Hospital of Nanjing Medical University, Nanjing 210000, PR China; b Clinical Medical Research Center, Jiangsu Province (Suqian) Hospital, Suqian 223800, China; c Second Department of Neurosurgery, "Attikon" University Hospital, National and Kapodistrian University of Athens, Greece; d Deparment of Neurosurgery, Fondazione Policlinico Universitario Agostino Gemelli IRCCS, Università Cattolica del Sacro Cuore, Rome, Italy; e Istituto neurologico Carlo Besta, Milan, Italy; f Department of neurosurgery, Jiangsu Province（Suqian）Hospital, Suqian, China

**Keywords:** Glucose-6-phosphate isomerase (GPI), Pan-cancer, Tumorigenesis, Biomarker, Bioinformatics, Immunotherapy

## Abstract

Glucose-6-phosphate isomerase (GPI) is a key metabolic enzyme implicated in tumorigenesis, yet its pan-cancer role and clinical significance remain incompletely understood. This study aimed to systematically characterize GPI expression, genetic alterations, clinical relevance, and biological functions across cancers, and validate its pro-oncogenic effects.We employed bioinformatics tools (GEPIA2, SangerBox 3.0, cBioPortal, TIMER 2.0, etc.) to analyze GPI expression, genetic alterations, and clinical relevance across multiple cancer types. In vitro experiments were conducted to validate its pro-oncogenic effects in LGG cells.Bioinformatics analyses showed that GPI was upregulated in 29 cancers and downregulated in 3 cancers. High GPI expression was associated with advanced stage, metastasis and poor prognosis. Genetic alterations positively regulated GPI expression, which was linked to cancer stemness, RNA modification, metabolic pathways and tumor immune microenvironment, and could predict chemotherapy response. In vitro experiments confirmed that GPI overexpression promoted LGG cell proliferation, colony formation and invasion, while knockdown inhibited these phenotypes. The correlation between DNA repair and GPI expression is interconnected through metabolic and genetic instability pathways, requiring further investigation.GPI acts as a pan-cancer pro-oncogenic factor and a promising diagnostic, prognostic, and therapeutic biomarker. Further research on its molecular mechanisms, particularly with DNA repair, will inform novel cancer treatment strategies.

## Introduction

Cancer remains a leading cause of global mortality, driven by complex dysregulations in metabolic, genetic, and immune pathways [1]. Identifying key molecular biomarkers and therapeutic targets is essential to improve clinical outcomes. Glucose-6-phosphate isomerase (GPI) is encoded by a member of the glucose phosphate isomerase protein family and is a conserved moonlighting protein, identified as such due to its ability to perform mechanistically distinct functions. Intracellularly, in the cytoplasm, it acts as a glycolytic enzyme that catalyzes the rate-limiting step in glycolysis, mediating the interconversion of glucose-6-phosphate and fructose-6-phosphate [1]. Extracellularly, this protein (also known as neuroleukin) functions as a neurotrophic factor that promotes the survival of skeletal motor neurons and sensory neurons, and as a lymphokine that induces immunoglobulin secretion. Additionally, based on its additional role as a tumor-secreted cytokine and angiogenic factor, it is also referred to as autocrine motility factor. Beyond these functions, GPI is also involved in cell migration and invasion, and its dysregulated expression has been reported in specific cancers, such as lung adenocarcinoma (LUAD) [2].

Existing studies have demonstrated GPI over-expression in tumors like lung and breast cancer, correlating with poor prognosis and advanced clinical stages [3]. However, these findings are limited to individual cancer types, lacking a systematic pan-cancer perspective. Critical gaps remain: the genetic and epigenetic mechanisms regulating GPI in diverse tumors are unclear, its association with cancer stemness (a key driver of therapy resistance and recurrence [4]) has not been comprehensively explored, and its role in shaping the tumor immune microenvironment requires validation. Additionally, while GPI's metabolic function suggests potential links to treatment responses, its utility as a predictive biomarker for chemotherapy or immunotherapy remains unconfirmed across cancers [5].

Cancer stem cells rely on metabolic reprogramming (e.g., glycolysis, glutamine metabolism) to maintain self-renewal and drug resistance [7], and GPI's position in glycolysis hints at possible involvement in stemness regulation. Moreover, GPI-related pathways may intersect with immune checkpoint signaling, as observed in LUAD, where GPI correlates with immune cell infiltration [8]. This study addresses these gaps by integrating multi-omics data from TCGA, GTEx, and other databases to perform a pan-cancer analysis of GPI [6]. We systematically evaluated GPI's expression patterns, genetic/epigenetic regulation, functional networks (metabolism, stemness, immunity), and clinical relevance (prognosis, treatment response). The findings will clarify GPI's multifaceted role in tumor biology and assess its potential as a clinical biomarker, laying the groundwork for personalized cancer therapy.

## Methods

### GPI protein and mRNA expression in normal tissues and cancers

The protein expression overview of GPI is downloaded from The Human Protein Atlas(TPHA) website(https://www.proteinatlas.org/). Then the GPI mRNA expression data and overview from the Genotype Tissue Expression (GTEx) are also downloaded from The Human Protein Atlas(TPHA) website. Type "GPI" into the search box on the webpage, then click the "search" button. Next, click the "Glucose-6-phosphate isomerase" tab and find "tissue RNA expression" under "protein summary",then we can obtain the protein and mRNA expression overview of GPI.The GPI mRNA expression data was obtained by navigating to the "DATA" section of the website, where the relevant RNA expression dataset was identified and subsequently downloaded from the "DOWNLOADABLE DATA" section. The result of the different mRNA expression level of the GPI in normal tissues and tumor tissues came from Sangerbox3.0(http://www.sangerbox.com)[9].

### The expression of GPI at different stages of cancer

The expression of GPI at different stages of cancer came from the "clinical staging and gene expression analysis" in Sangerbox3.0(ENSG00000105220(GPI)).And the data was downloaded from the unified standardized pan-cancer dataset in the UCSC (https://xenabrowser.net/) database.

### Gene mutation and expression analysis

The alteration frequency of GPI in 32 cancers was obtained from cBioPortal(https://www.cbioportal.org/). We log into the website, enter "GPI" in the search box, select "gene GPI", and then choose the section "Cancers Types Summary". And we got the percentage of patient samples with GPI mutations from TIMER2.0.The mRNA expression values of GPI and its corresponding copy-number alteration values were analyzed using the SangerBox portal. Correlations of GPI expression and somatic mutations were obtained from the Comprehensive Analysis on Multi-Omics of Immunotherapy in Pan-cancer (CAMOIP) by selecting the "Mutational Landscape" section and the "gene expression" section [10].

### Survival prognosis analysis

Using the SangerBox online tool and following the website instructions, the overall survival, disease-specific survival, disease-free survival, and progression-free survival of GPI in various tumor types in the TCGA and GTEx database were evaluated through forest plots. Overall survival map and the Kaplane-Meier survival plots were generated using GEPIA2 by selecting the "Expression Analysis" and "Survival Analysis" sections [11]. High-expression and low-expression cohorts of GPI were obtained through the expression threshold of the GPI-high (top 25% expression) and GPI-low (bottom 75% expression) subgroups stratified by GEPIA2 quantile cutoff.

### Correlation analysis of GPI mRNA expression with DNA methylation

The scatter plots showing the correlation between DNA methylation of the GPI gene and mRNA expression were downloaded from the cBioPortal website [12].After we enter the cBioPortal website, we input the name of the tumor(TCGA,Firehose Legacy) in the search box. After reaching the next page, we input “GPI” in the small search box at the top right corner. Finally, we select the “Plots” section to obtain the relevant data.

### GPI co-expression gene enrichment analysis

The co-expressed genes of GPI and their corresponding Pearson correlation coefficients were downloaded from the cBioPortal website too. We select the “Co-expression” section to obtain the relevant data. Using the GEPIA2, by selecting the "Multiple Gene Comparison" option, the heat map of GPI-positive genes can be visually displayed. GPI positively correlated genes with Spearman's Correlation > 0.3 were selected to perform Gene Ontology (GO) term enrichment and Kyoto Encyclopedia of Genes and Genomes (KEGG) enrichment analysis using SangerBox portal. The SangerBox was used to visually present Venn diagrams, GO string diagrams, and GO/KEGG pathways. The experimentally verified protein-protein interaction network of GPI was analyzed using the online tool String(https://cn.string-db.org/)[13] scatter plot created through GEPIA 2.0 visually presents the correlation between GPI and the top five co-expressed genes.

### Analysis of immune infiltration and immune-related genes

Through the SangerBox website, using Person's R correlation coefficient, the expression of GPI was evaluated in relation to the stroma score, immune score, ESTIMATE score, or the proportion of tumor-infiltrating immune cells (including CD4 + T cells, CD8 + T cells, B cells, neutrophils, macrophages, and dendritic cells) within tumors in different cancer types. The correlation between GPI expression and various immune checkpoint blockade-related genes across multiple cancer types was generated by the SangerBox website.

Correlation coefﬁcient (Pearson’s R) between GPI expression and tumor mutation burden (TMB), microsatellite instability (MSI), or neoantigens was downloaded and analysed from the SangerBox portal. Furthermore, through the SangerBox website, the correlation between the expression of GPI and immune regulatory genes (including immunosuppressants and immunostimulants) in different tumor samples was also evaluated. These gene data were obtained from the TCGA + GTEx. Furthermore, using TISIDB, the correlation between GPI and chemokines or major histocompatibility complex (MHC) was also evaluated [10], [14].TISIDB integrates a variety of data resources in the field of tumor immunology and can be accessed by selecting the "Chemokines" and "Immune Modulators" sections. By selecting the "immunotherapy" option in TISIDB, the expression levels of GPI in both responders and non-responders to immunotherapy were also obtained.

### Evaluation of the therapeutic response to GPI, as well as compound research

The TCGA drug response analysis of GPI in various types of cancers was used the "Drug analysis" section in GEPIA3(gepia3.bioinfoliu.com) [15].Correlation analysis between the expression level of GPI and the overall survival period of patients treated with docetaxel and capecitabine was also used the "Drug analysis" section in GEPIA3.Furthermore,the relationship between GPI and drug sensitivity was conﬁrmed using RNAactDrug [16],which serves as a comprehensive resource for drug sensitivity and RNA molecular correlations, selecting drugs with an FDR < 0.05.

### Cell lines and cell culture

The human LGG cell lines HS683 and GBM cell lines U87 were obtained from the American Type Culture Collection (ATCC). HS683 cells are cultured in DMEM supplemented with 10% fetal bovine serum and maintained in a growth state in a 37°C, humidified incubator supplemented with 5% CO2. U87 cells grown in Lebovitz L-15 medium supplemented with 10% fetal bovine serum and maintain their growth state under 37°C conditions.

### Western blotting

Extract the total protein from each group of cells. After quantification by the BCA method, take an equal amount of protein for SDS-PAGE electrophoresis. Transfer it onto a PVDF membrane, and after blocking, add the GPI antibody (diluted 1:1000) and the internal reference α-Tubulin antibody (diluted 1:5000). Incubate at 4℃ overnight. The next day, add the fluorescent secondary antibody (diluted 1:2000), and after developing, quantify the gray value of the bands using ImageJ software.

### Cell proliferation ability experiment: CCK-8 method

The cells in each group were inoculated into 96-well plates at a density of 5 × 10 ³ cells per well. On the 1st, 3rd, and 5th days of culture, 10 μL of CCK-8 reagent was added to each well. After continued culture for 2 h, the absorbance value at 450 nm wavelength was detected using an enzyme detector to reflect the cell viability.

### Colony formation assay

Dilute each group of cells to 1 × 10 ³ cells/mL. Take 2 mL and inoculate them into 6-well plates. After conventional culture for 14 days, discard the culture medium, fix with 4% paraformaldehyde for 15 min, stain with crystal violet for 30 min, rinse with running water and dry, then count the number of clones with a diameter > 0.5 mm.

### Transwell invasion assay and analysis of fluorescent invasion regions verification

In the Transwell experiment, the upper chamber was coated with Matrigel matrix gel, and 5 × 10⁴ cells were inoculated into the upper chamber. The lower chamber was filled with culture medium containing 20% fetal bovine serum. After 24 h of culture, the cells on the lower chamber surface were fixed and stained. Five random fields were selected to count the number of invasive cells. In the fluorescence invasion area analysis, the cells were labeled with CFSE fluorescent dye and inoculated into the invasion chamber. After 24 h of culture, the invasion area was observed under a fluorescence microscope and quantified using ImageJ software.

### Statistical Analysis

All pan-cancer multi-omics data were derived from TCGA Firehose Legacy, GTEx v8, cBioPortal, TIMER2.0, GEPIA2, GEPIA3, SangerBox 3.0, TISIDB, CAMOIP, RNAactDrug and STRING databases; for each analysis, we recorded the cancer type, total sample size, counts of tumor and normal subgroups, database release version and data download date, all raw RNA-seq data from TCGA and GTEx were uniformly normalized by log2(TPM+1), and the ComBat algorithm was applied to eliminate batch effects between TCGA tumor cohorts and GTEx normal cohorts. For survival grouping, stratification in GEPIA2 overall survival analysis was clearly defined as GPI high = top 25% expression and GPI low = bottom 75% expression to remove ambiguous descriptions, log-rank test was used for all Kaplan–Meier survival analyses, and Cox proportional hazard regression was conducted to calculate hazard ratios (HR) with 95% confidence intervals (95%CI). Pearson correlation analysis was adopted for pairwise correlation of continuous gene expression, Student’s *t*-test and one-way ANOVA were used for two-group and multi-group expression comparisons respectively, and Wilcoxon rank-sum test was performed for non-normally distributed data. FDR (Benjamini–Hochberg) correction was applied to all pan-cancer screening analyses covering 32 cancer types, dozens of immune markers, RNA-modification genes and multiple survival endpoints, both raw P-values and FDR-adjusted P-values were reported in all figures, tables and supplementary materials, and analyses without FDR adjustment were explicitly labeled as exploratory. The software versions used in this study included GEPIA2 (v2.0), GEPIA3 (v3.0), SangerBox (v3.0), TIMER2.0, cBioPortal (v3.6.18), TISIDB, CAMOIP and RNAactDrug, and STRING v11.5 was utilized for protein-protein interaction network analysis. The statistical significance threshold was set as adjusted P < 0.05, and correlation coefficient |R| > 0.3 was defined as moderate correlation for co-expression screening. A standalone Data Availability Statement was added at the end of the manuscript, containing all database accession links, raw data download URLs and complete screenshots of analysis parameters exported from each online portal, and all customized analysis scripts generated by SangerBox were uploaded as Supplementary File S4 to improve research reproducibility.

## Results

### The expression of GPI changes along with the progression stage in multiple types of cancer

**Fig. 1:**
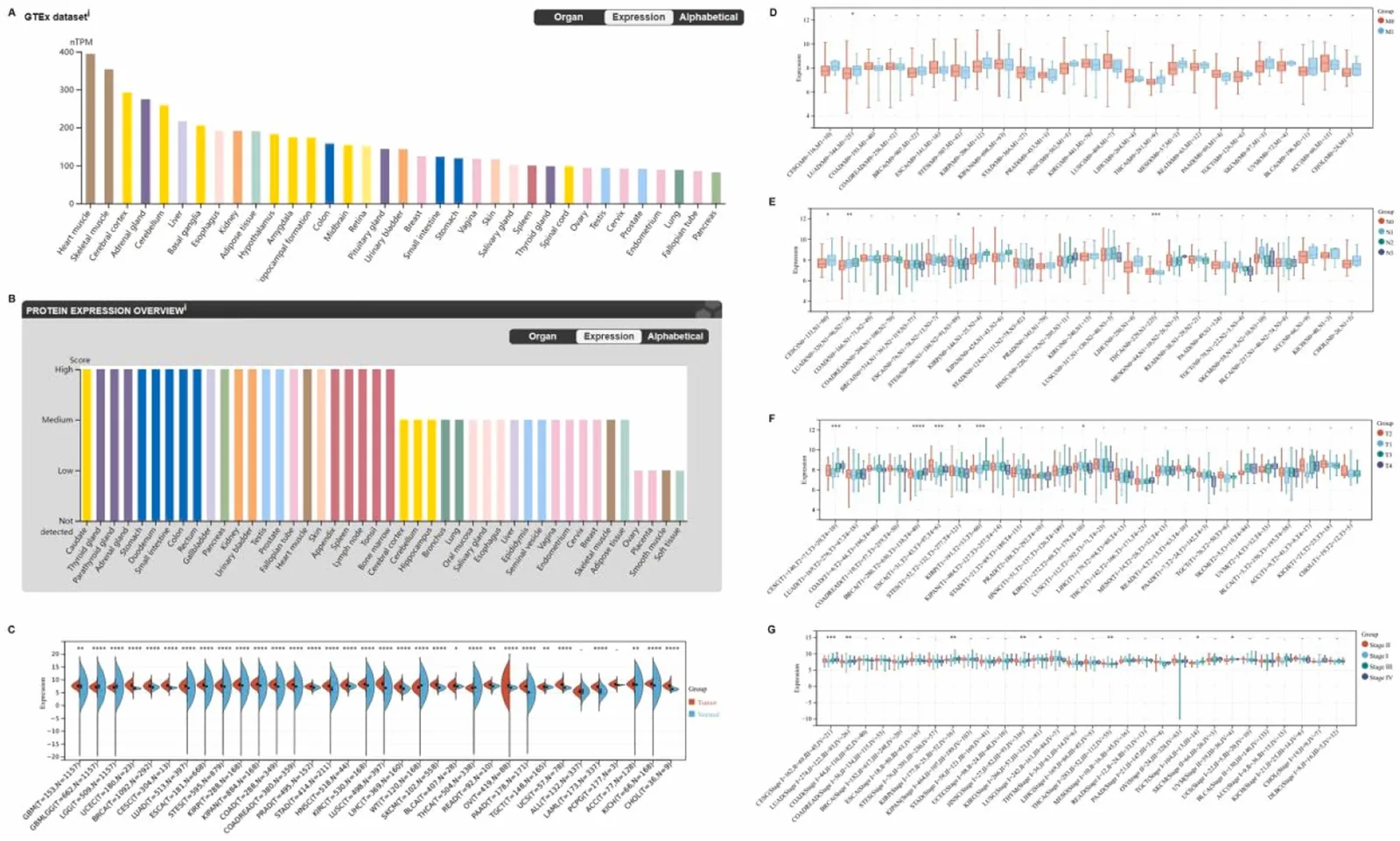
The mRNA expression levels of GPI in human pan-cancer. (A) GPI mRNA expression in various human normal tissues. Genotype Tissue Expression (GTEx) data for GPI mRNA in human normal tissues were downloaded from The Human Protein Atlas (THPA). (B) GPI protein expression overview in various human normal tissues from The Human Protein Atlas (THPA). (C) GPI mRNA expression levels in tumor tissues were compared with normal tissues across 34 cancer types from Sangerbox3.0. (D) GPI mRNA expression in the M staging of tumors. (E) GPI mRNA expression in the N staging of tumors. (F) GPI mRNA expression in the T staging of tumors.(G) GPI mRNA expression in the different staging of tumors.*p < 0.05, **p < 0.01, ***p < 0.001, ****p < 0.0001; ns, no significance.

We found that, in normal tissues, the mRNA expression of GPI is highest in heart muscle, followed by skeletal muscle, cerebral cortex, adrenal gland, and so on. This indicates that GPI is widely expressed in these organs and tissues and may play a basic role (Fig. 1A). Based on the data for mRNA expression of GPI, the heart muscle ranks among the high-level group, while the skeletal muscle and cerebral cortex rank among the medium-level group (Fig. 1B). Next, we wonder if there are any differences in the expression of GPI in normal tissues or organs compared to its expression in tumor tissues. In 29 types of tumors, we observed significant up-regulation such as GBM, UCEC, BRCA (Fig. 1C). At the same time, we observed significant down-regulation in three types of tumors including LGG (Fig. 1C). These results indicates that GPI plays a certain role in the occurrence of tumors and can be used as a potential diagnostic biomarker for these cancer types.

We further analyzed the expression levels of GPI at different stages of cancer using the Sangerbox 3.0. From stage M0 to stage M1 of tumors, we observed a significant increasement in LUAD, suggesting that GPI is important for distant organ metastasis in LUAD (Fig. 1D). From stage N0 to stage N3 of tumors, we found significant increasement in 4 types of tumors, including CESC, LUAD, STES, and THCA, indicating that GPI is significative for lymphatic system metastasis (Fig. 1E). From stage T1 to stage T4 of tumors, we noticed significant differences in 6 types of tumors, including CESC, BRCA, ESCA, STES, KIRP, and KIRC (Fig. 1F). From the overall staging of the tumors, we acquired a gradual increase in GPI mRNA levels across different cancer stages in HNSC, while a gradual decrease in CESC, LUAD, TGCT, and UVM (Fig. 1G). This further confirms that GPI is associated with the progression of these cancers.

In conclusion, the GPI plays an oncogenic role and is associated with the occurrence, development and metastasis of certain cancer types.

### Alterations in the GPI gene are associated with the occurrence and development of various types of cancers

**Fig. 2:**
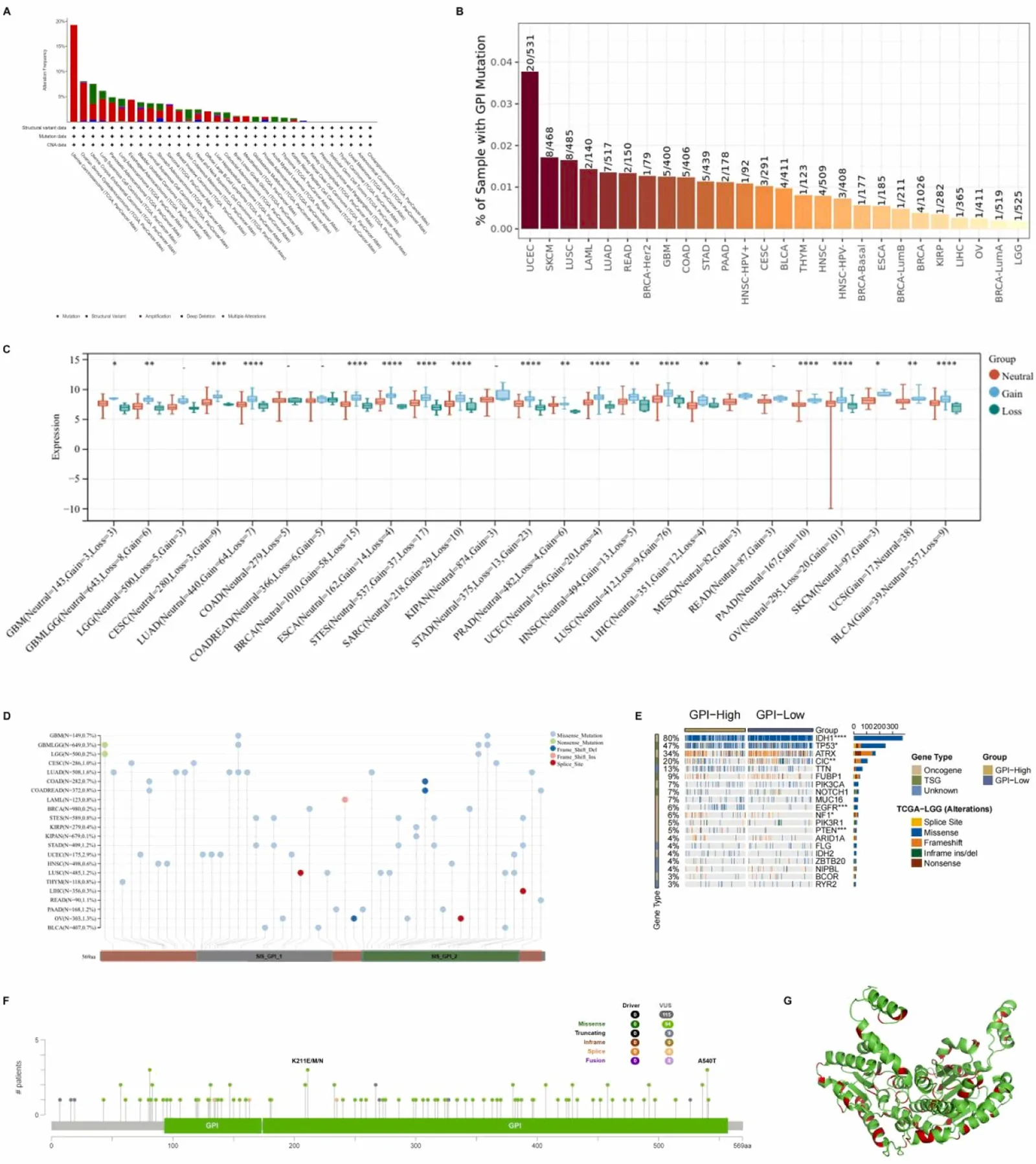
Distinct genomic profile analysis of GPI in various cancers. (A) Alteration frequency of GPI across various cancer types. The data were obtained from the cBio Cancer Genomics Portal (cBioPortal) database, showing the percentage of samples with mutations, structural variants, amplifications, and deep deletions in GPI across different cancer types. (B) Mutation frequency of GPI in different cancer types. The data from Tumor Immune Estimation Resource 2.0 (TIMER2.0) reveals the percentages of mutations of GPI in various cancers. (C) The mutation landscape of GPI in different cancer types. The results were obtained from SangerBox comparing neutral, gain, and loss alterations in various cancers. (D) Mutation types and distribution in GPI across cancers. The data was analyzed using the SangerBox portal, showing the percentages of missense mutations, nonsense mutations, and splice sites across cancer types. (E) The relationship between GPI expression and somatic mutations in LGG cancers. The data from Comprehensive Analysis on Multi-Omics of Immunotherapy in Pan-cancer (CAMOIP) show the percentages of oncogene, tumor suppressor gene, and unknown gene types, and show the numbers of different mutation types such as splice site, missense, frameshift, inframe insertion/deletion, and nonsense mutations in the GPI-high and GPI-low groups. (F)Protein domain analysis of GPI mutations. The mutation map generated from cBioPortal shows mutation types in the GPI domains. (G) The protein structure diagram of GPI and the amino acid mutation sites. The data was downloaded from the RCSB PDB(https://www.rcsb.org/). The red part in the figure represents the common mutation sites of GPI in various cancers.*p < 0.05, **p < 0.01, ***p < 0.001, ****p < 0.0001; ns, no significance.

In addition to gene expression, the occurrence, development and treatment outcomes of cancer (such as survival rate and disease recurrence) are also influenced by genetic variations and mutations. Understanding these alterations is helpful in clarifying the mechanisms of cancer development and disease progression, and it also plays a crucial role in guiding the diagnosis and treatment of cancer [7], [8]. Thus, we analyzed the genetic profile of GPI in cancer tissues. UCS (19.3%), OV (7.53%), ESCA (4.4%), LUSC(4.31%), PAAD(3.8%), UCEC(3.21%), Sarcoma(SARC)(3.14%), CESC(2.69%), LUAD(2.47%), BLCA(2.19%), DLBC(2.08%), BRCA(2.03%), STAD(1.59%), HNSC(1.15%), MESO (1.15%), and LIHC (1.08%) had a higher rate of GPI gene amplification frequency, while no one had a higher rate of GPI gene deep deletion frequency (Fig. 2A). Further analysis showed that gain copy number of GPI is associated with higher mRNA expression levels of GPI in 20 cancer types, including GBM (Fig. 2C), suggesting that GPI mRNA expression is mainly due to copy number alterations.

Using cBioPortal, we obtained the alteration frequency of GPI in 32 cancers. And UCEC (3.97%) had the highest rate of GPI gene mutations, followed by SKCM(2.04%), LUSC(1.64%), LUAD(1.59%), STAD(1.36%),COAD(1.18%),PAAD(1.09%),GBM(1.03%),CESC(1.01%),Acute Lymphoblastic Leukemia(ALL)(1%), BLCA(0.97%), THYM(0.81%), HNSC(0.76%), LIHC(0.54%), BRCA(0.37%), KIRP(0.35%), LGG(0.19%), and OV(0.17%) (Fig. 2A). Furthermore, we investigated the mutation rate of the GPI gene on the TIMER 2.0, and obtained results that were almost identical to the aforementioned findings (Fig. 2B). We further investigated the types and sites of mutations in GPI, and found that missense mutations were the main type of GPI mutations in cancer (Fig. 2D). The mutation sites frequently occur within the PGI domain (Fig. 2E). The missense hotspot mutations K211E/M/N and A540T were located within the conserved PGI catalytic domain of GPI protein, which mediates glycolytic enzymatic activity. It suggests that the mutations of GPI, especially the mutation at the K211E/M/N and A540T, could be a diagnostic biomarker.

Then, the correlation between GPI expression and specific genomic characteristics, such as somatic mutations, was analyzed. We found that GPI was strongly associated with somatic mutations in multiple cancers. The GPI-high group showed high frequency of somatic mutations in PETN (58%), PIK3CA(49%), TTN (45%), ARID1A (44%) in UCEC, and IDH1 (80%),TP53 (47%), ATRX (34%), CIC (20%), TTN (13%),FUBP1 (9%), PIK3CA (7%)in LGG (Fig. 2F). Taken together, these results suggested that GPI gene alterations may regulate the initiation, growth, and progression of various cancers and can serve as a potential diagnostic biomarker.

### The expression of GPI is associated with the prognosis of various tumors

**Fig. 3:**
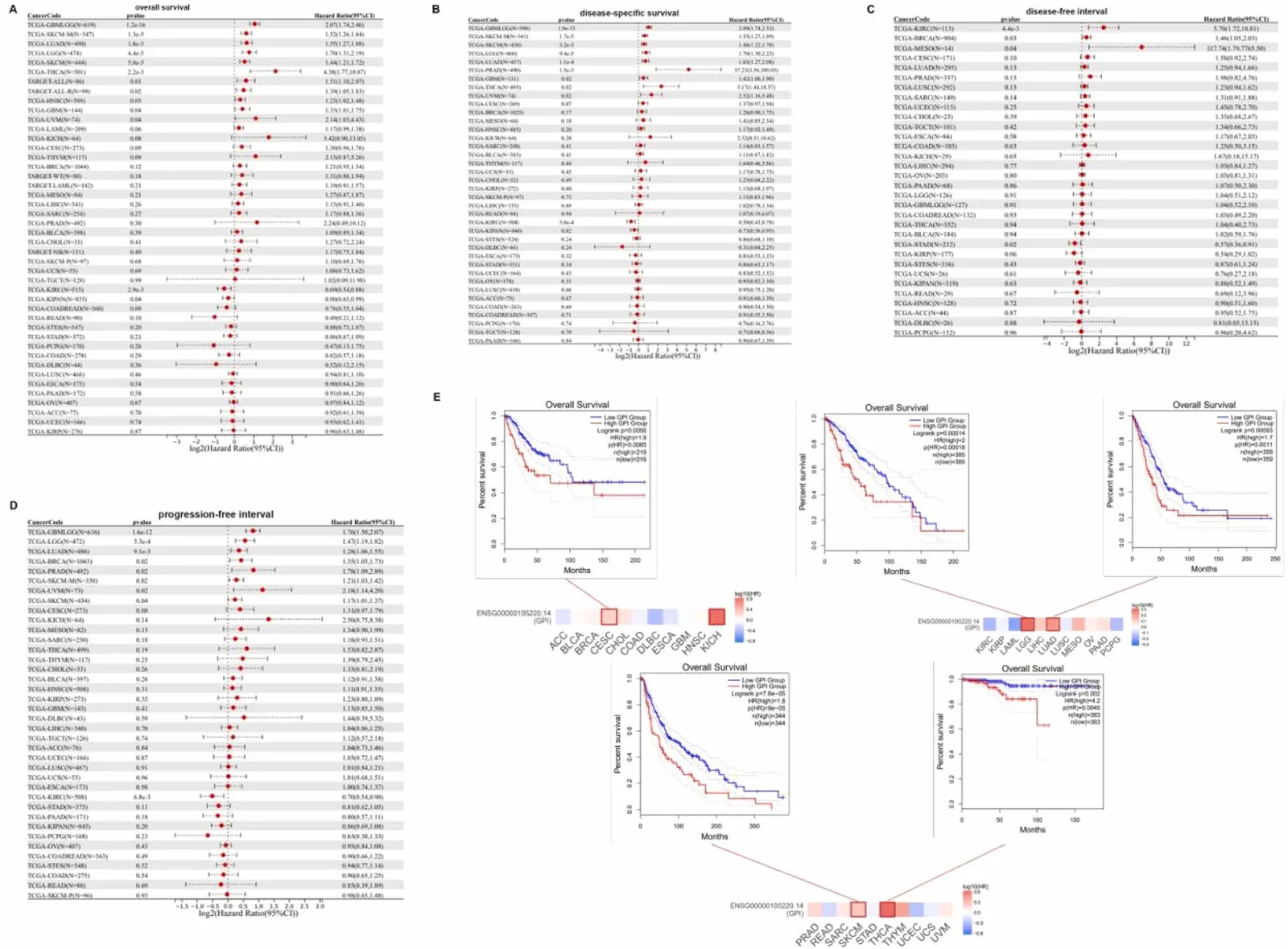
Correlation analysis between GPI expression and survival prognosis in various cancers. (A-D) The forest plot presents the hazard ratios (HR) of GPI expression in different types of cancers. These data are from SangerBox and show the correlation between GPI expression and overall survival rates(A), disease-specific survival rates(B), disease-free interval rates(C), progression-free interval rates(D) across various cancer types. A p-value less than 0.05 is considered significant. (E) Overall survival graphs and Kaplan-Meier survival curves for different cancer types. These results were generated using the GEPIA2.0 online tool. The survival heatmap above shows the hazard ratio (HR) in logarithmic 10-fold units; red indicates a higher HR, and blue indicates a lower HR. The cancer types highlighted in red boxes on the heatmap indicate statistical significance, with a p-value less than 0.05. In the Kaplan-Meier plot, the blue and red lines represent patients with low and high GPI expression levels, respectively. Each graph shows the log-rank p-value and hazard ratio (HR).

The gene expression pattern can predict the prognosis of patients, such as overall survival, disease-specific survival, disease-free interval, and progression-free interval [17]. The above research results indicated that GPI had the potential to be further studied as a prognostic biomarker. Therefore, we compared the survival situations of cancer patients with high or low expression of GPI. We generated survival forest plots and found that higher GPI expression was significantly associated with worse patient overall survival in GBMLGG (HR = 2.07, p = 1.2e-16), SKCM-M(HR = 1.52, p = 1.3e-5), LUAD (HR = 1.55, p = 1.8e-5), LGG (HR = 1.70, p = 4.4e-5), SKCM (HR = 1.44, p = 5.0e-5), THCA (HR = 4.38, p = 2.2e-3), HNSC (HR = 1.23, p = 0.03), GBM (HR = 1.33, p = 0.04) and UVM (HR = 2.14, p = 0.04). In contrast, higher GPI expression was significantly associated with lower risk of death in KIRC (HR = 0.69, p = 2.9e-3) and KIPAN (HR = 0.80, p = 0.04) (Fig. 3A). Furthermore, disease-specific survival analysis indicated that GBMLGG (HR = 2.09, p = 1.9e-15), SKCM-M (HR = 1.55, p = 1.7e-5), SKCM (HR = 1.48, p = 5.2e-5), LGG (HR = 1.70, p = 9.4e-5), LUAD (HR = 1.63, p = 1.1e-4), PRAD(HR = 37.23, p = 1.5e-3), GBM(HR = 1.42, p = 0.02), THCA(HR = 5.17, p = 0.02), UVM(HR = 2.52, p = 0.02), KIRC(HR = 0.58, p = 3.6e-4), and KIPAN (HR = 0.73, p = 0.02) had significantly stronger association between GPI and patient survival (Fig. 3B). In GBMLGG, SKCM-M, SKCM, LGG, LUAD, PRAD, GBM, THCA, and UVM, patients with higher expression levels of GPI have a higher risk of death. In contrast, in KIRC and KIPAN, patients with lower expression levels of GPI have a higher risk of death. And disease-free survival analysis showed that BRCA (HR = 1.46, p = 0.03), KIRC (HR = 5.70, p = 4.4e-3), MESO (HR = 117.74, p = 0.04), and STAD (HR = 0.57, p = 0.02) had significantly stronger association between GPI and patient survival (Fig. 3C). In BRCA, KIRC, and MESO, patients with higher expression levels of GPI have a higher risk of death, while it has a high risk of death in STAD with lower expression levels of GPI. Next,progression-free survival revealed that GBMLGG (HR = 1.76, p = 1.6e-12), LGG (HR = 1.47, p = 3.3e-4), BRCA (HR = 1.35, p = 0.02), LUAD (HR = 1.28, p = 9.1e-3), PRAD (HR = 1.78, p = 0.02), SKCM (HR = 1.17, p = 0.04), SKCM-M (HR =1.21, p = 0.02), UVM (HR = 2.18, p = 0.02), and KIRC(HR = 0.70, p = 6.8e-3) had significantly stronger associations between GPI and patient survival(Fig. 3D). In GBMLGG, LGG, BRCA, LUAD, PRAD, SKCM, SKCM-M, and UVM,patients with higher expression levels of GPI have a higher risk of death,whereas it has a high risk of death in KIRC with lower expression levels of GPI(Fig. 3D).

Next, we generated Kaplane-Meier survival curves to demonstrate the correlation between the expression of GPI and the overall survival rate of patients with different types of cancers. High GPI expression is associated with worse patient survival for CESC (HR = 1.9, p = 0.0056),KICH (HR = 6.9, p = 0.0017),LGG (HR = 2, p = 0.00014),LUAD (HR =1.7, p = 0.00093),SKCM (HR =1.8, p = 7.6e-5), and THCA (HR = 4.2, p = 0.002)(Fig. 3E).

Overall, the significant associations between GPI expression and patient prognosis strongly suggest that GPI could be a potential prognostic biomarker for various cancers.

### Correlation analysis of GPI mRNA expression with RNA modifications and DNA methylation in various types of cancer

**Fig. 4:**
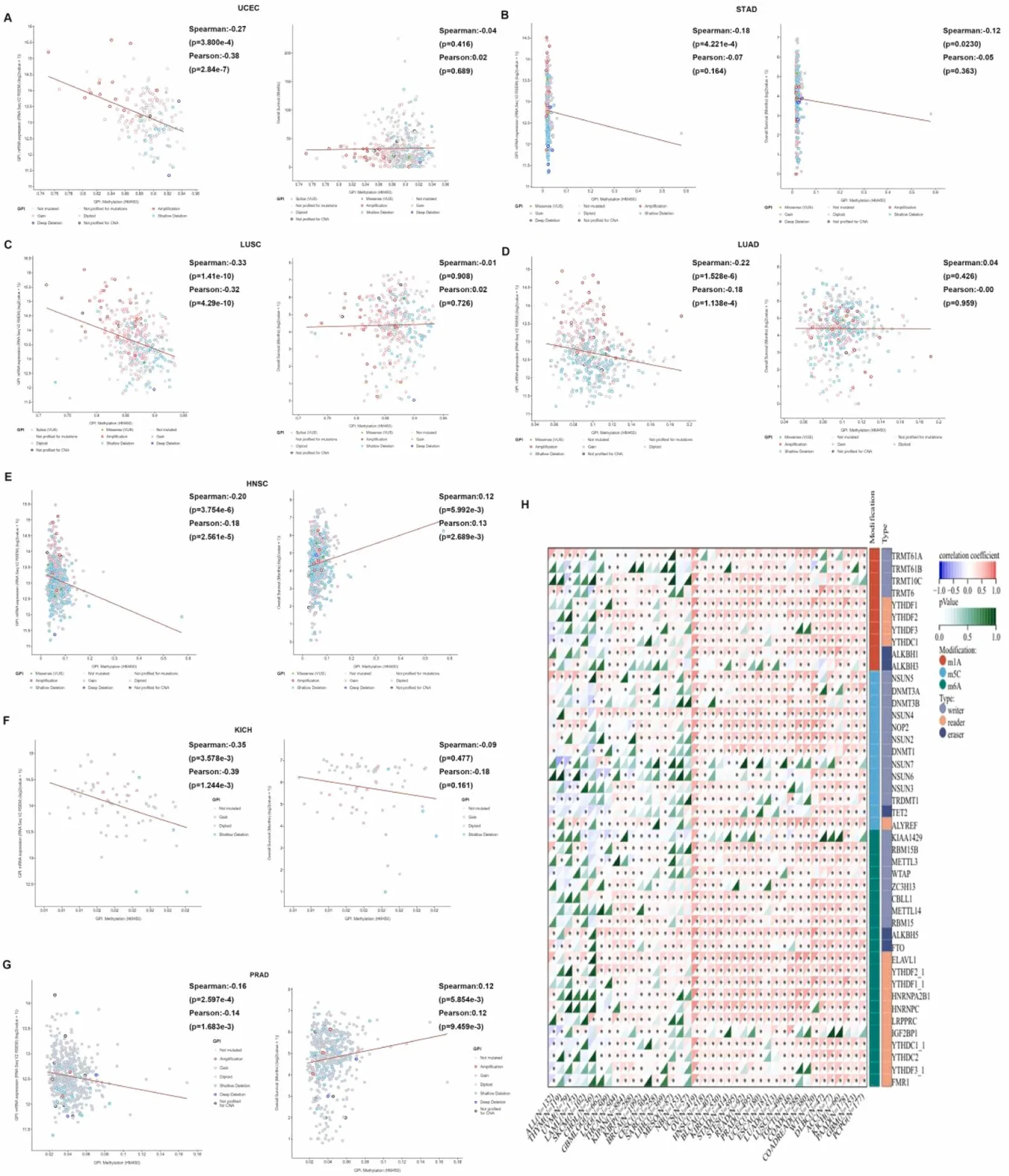
Correlation analysis between GPI and GPI methylation level or RNA modifications. (A-G) Correlation analysis of GPI methylation level with the overall survival period of patients and the correlation between the methylation level of GPI and the mRNA expression level in UCEC(A), STAD(B), LUSC(C), LUAD(D), HNSC(E), KICH(F), PRAD(G). (H)This heatmap illustrates the correlation between the expression of GPI and various genes related to RNA modifications (writers, readers, and deleters) in different cancer types. The data is presented in the form of correlation coefficients, with colors ranging from red (positive correlation) to blue (negative correlation). The colored bars on the right indicate the types of RNA modifications (m1A, m5C, and m6A). The p-values for each correlation are shown on the heatmap, with significant correlations marked by asterisks, and deep green indicates no significant correlation.

When the expression level of GPI is high, its RNA expression pattern is similar to that of cancer stem cells, suggesting that GPI is related to epigenetic modifications. Epigenetic modifications, such as RNA modifications (which represent a new frontier in this field), have significant implications for the development and treatment of cancer [18]. Therefore, we used the SangerBox online tool to analyze the correlation between the expression of GPI mRNA and the RNA-modifying enzymes that catalyze m1A, m5C, and m6A modifications. These RNA modifications play a crucial role in cancer, as they can regulate gene expression, mRNA stability, translation, and the progression of tumor development [18].According to Fig. 4H, the expression of GPI mRNA is significantly positively correlated with the RNA modification enzymes related to m1A, m5C and m6A in OV, HNSC, BLCA, KIRC, STAD, STES, READ, PRAD, UCEC, ESCA, LUAD, LUSC, TGCT, COAD, COADREAD, WT, DLBC, ACC, KICH, PAAD,GBM,and PCPG, suggesting that m1A, m5C, and m6A RNA modifications may affect the gene expression related to the progression of these cancer tumors, and this is especially true when the expression of GPI increases. In contrast, the expression of GPI mRNA is significantly negatively correlated with the RNA modification enzymes related to m1A, m5C and m6A in ALL, THYM, UVM, and CHOL.Overall, these results suggested that GPI may be a potential biomarker for identifying tumors with high stem cell characteristics, and can serve as a therapeutic target to inhibit these aggressive tumor features.

DNA methylation is a crucial epigenetic modification, which refers to the process where methyl groups are added to specific bases of DNA (typically cytosine in CpG dinucleotides) under the catalysis of DNA methyltransferases (DNMTs). It plays a central role in gene expression regulation, cell differentiation, and disease occurrence [19].Tumor cells generally exhibit "methylation imbalance" - local hyper-methylation (silencing of tumor suppressor gene promoters) coexists with global hypomethylation (increased genomic instability). Abnormal DNA methylation is an early event in the occurrence of cancer and can serve as an early diagnostic marker [20].Utilizing the cBioPortal website, we analyzed the promoter methylation levels of GPI across various cancer types. As shown in Fig. 4A-G, the DNA methylation level of the GPI was significantly negatively correlated with the mRNA expression level in UCEC, STAD, LUSC, LUAD, HNSC, KICH, and PRAD. The methylation level of the GPI was positively correlated with the overall survival period of the patients in HNSC and PRAD. In contrast, the methylation level of the GPI was negatively correlated with the overall survival period of the patients in STAD. And there was no significant correlation between the methylation level of the GPI and the overall survival period of the patients in KICH, UCEC, LUSC and LUAD.

### Enrichment analysis of GPI co-expressed genes in various tumors

**Fig. 5:**
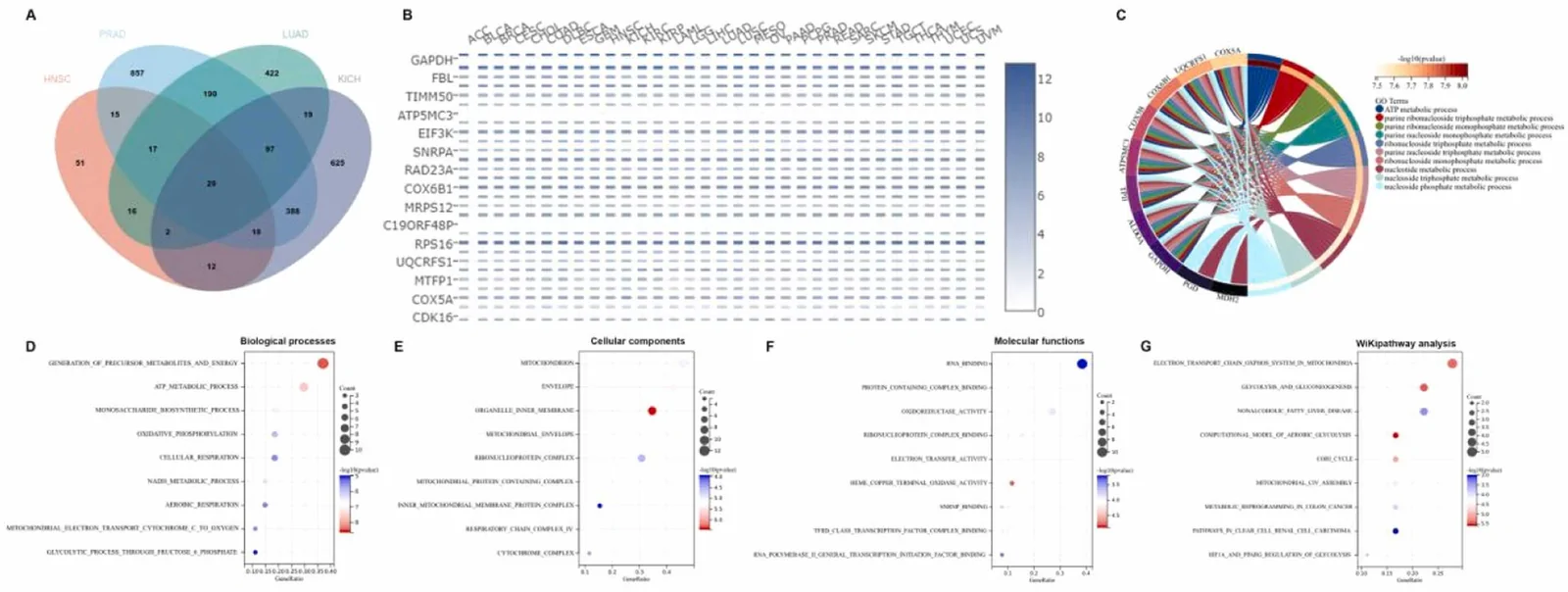
Enrichment analysis of co-expressed genes of the GPI gene in various tumors. (A) A Venn diagram was generated using SangerBox, which illustrated the overlapping situation of GPI co-expressed genes in four different tumor types: LUAD (green), KICH (purple), HNSC (red), and PRAD (blue).The numbers in the figure indicate the quantities of common and unique genes that are co-expressed with GPI in these tumors. (B)A heatmap was generated using Gene Expression Profiling Interactive Analysis 2.0 (GEPIA2.0), which displayed the expression levels of 29 genes co-expressed with GPI in different types of cancers. The color gradation from light blue to dark blue indicates the gradual increase in gene expression levels.(C) A Gene Ontology (GO) Chord plot was generated using SangerBox, displaying the enrichment of the 29 GPI coexpressed genes. The biological process pathways are highlighted in the form of colored strips, and their widths are proportional to the number of enriched genes.(D-G) The bubble plots were generated from SangerBox, representing the results of the Gene Ontology (GO) and Kyoto Encyclopedia of Genes and Genomes (KEGG) pathway enrichment analysis of 29 GPI genes that are co-expressed in tumors.(D) Biological processes associated with genes co-expressed with GPI.(E) Cellular components related to genes co-expressed with GPI.(F) Molecular functions of genes co-expressed with GPI.(G) KEGG pathway analysis shows significant enrichment. The size of each bubble corresponds to the number of genes involved, while color intensity indicates the significance level (P).

In order to understand the biological significance of GPI and its co-expressed genes, as well as their roles in cellular processes, and to clarify how GPI affects the development of cancer and the prognosis of patients, we conducted a KEGG density analysis of GPI and its co-expressed genes. Because GO and KEGG analysis is crucial for understanding the biological significance of genes and their roles in cellular processes [21], [22]. Since HNSC, KICH, LUAD, and PRAD all showed higher mRNA expression levels of GPI, and high levels of RNA modifications with worse patient survival from the results above, we would like to know if there is any overlapping expression of the GPI gene among these cancers, and whether these genes might be related to the fundamental processes associated with GPI in cancer. First, we downloaded the genes that co-expressed with GPI from the cBioPortal website for these 4 types of cancers, and performed Venn analysis using SangerBox online tools, then found that there were 29 overlapping genes among the 4 types of cancers analyzed(Fig. 5A). Next, we analyzed the mRNA expression levels of these 29 overlapping genes in various cancers. The heatmap revealed that 27 of the overlapping genes showed high expression in all the different types of cancers(Fig. 5B). This indicated that these genes may play a crucial role in the process of individual development and cancer progression. Then, through GO enrichment analysis, we found that these genes are related to processes such as generation of precursor metabolites and energy, ATP metabolic process, monosaccharide biosynthetic process, oxidative phosphorylation, cellular respiration, NADH metabolic process, aerobic respiration, mitochondrial electron transport cytochrome C to oxygen, and glycolytic process through fructose-6-phosphate(Fig. 5C,D).They were mainly located in mitochondria, envelopes, organelle inner membranes, ribonucleoprotein complex, respiratory chain complex Ⅳ,and cytochrome complex(Fig. 5E), with molecular functions of RNA binding, protein containing complex binding, oxidoreductase activity, ribonecleprotein complex binding, electron transfer activity, heme copper terminal oxidase activity, snRNP binding, TFⅡD class transcription factor complex binding, and RNA polymerase II general transcription initiation factor binding(Fig. 5F). In order to gain a deeper understanding of how these genes interact with the networks and pathways, we also conducted a KEGG enrichment analysis. These genes that are co-expressed with GPI are related to electron transport chain oxphos system in mitochondria,glycolysis and gluconeogenesis, nonalcoholic fatty liver disease, cori cycle, mitochondrial civ assembly, metabolic reprogramming in colon cancer, and pathways in clear renal cell carcinoma(Fig. 5G).

We identified the top 10 proteins interacting with GPI(Fig. S1A), after further analysis, the top 5 proteins with the most interactive with GPI are TPI1,G6PD,H6PD, MPI, and TKT.Next,we further analyzed the correlation between GPI and these 5 proteins in all types of cancers. It showed that G6PD showed weak positive correlation with GPI (R = 0.33, p < 2.2e-16); H6PD exhibited negligible yet statistically significant association (R = 0.053, p = 2.4e-07); MPI displayed weak positive correlation (R = 0.16, p < 2.2e-16); TKT had moderate-weak positive correlation (R = 0.27, p < 2.2e-16); and TPI1—the strongest link among the five—showed moderate positive correlation with GPI (R = 0.47, p < 2.2e-16)(Fig. S1B). This pattern aligned TPI1 (a key glycolytic enzyme functionally coordinated with GPI via shared glycolysis pathways) with the most robust transcriptional coordination with GPI, while H6PD (a pentose phosphate pathway enzyme) showed minimal co-regulation, and the remaining proteins exhibit weak to subtle co-expression links. TPI1 (Triosephosphate isomerase 1) plays a crucial role in tumor development by maintaining efficient glucose metabolism in tumor cells and regulating proliferation signaling pathways. It is one of the key metabolic enzymes that support the malignant phenotype of tumors. Therefore, GPI may exert its tumor-promoting effect through its interaction with TPI1.

In conclusion, these results indicated that the co-expressed genes of GPI are involved in important biological processes, particularly those related to cellular energy production and metabolism, and are likely to play a significant role in the survival and proliferation of cancer cells.

### GPI regulates tumor immune cell infiltration in various types of cancers and is closely associated with immune checkpoint blockade proteins, serving as a viable biomarker for tumor immunotherapy

The tumor microenvironment (TME) is composed of various immune cells, stromal cells, and extracellular components [23].Immune infiltration analysis is helpful in understanding the composition and functional status of these immune cells in the tumor microenvironment, as well as the influence of the tumor microenvironment on tumor growth and metastasis. StromalScore is an indicator used to quantify the content of stromal cells (including fibroblasts, endothelial cells, adipocytes, etc.) in the tumor microenvironment [24]. The higher the score, the greater the proportion of stromal cells in the tumor microenvironment. Furthermore, a higher stromal content is usually associated with stronger tumor growth characteristics, resistance to treatment, and poorer prognosis [25].ImmuneScore is a crucial indicator used to quantify the degree and activity of immune cell infiltration in the tumor microenvironment, reflecting the overall infiltration level and functional activity of immune cells in the tumor tissue [24].The higher the score, the more abundant the infiltration of immune cells in the tumor microenvironment and the stronger the immune response activity. A higher immune score is usually associated with a better response to immunotherapy and a better prognosis for the patients [26].ESTIMATEScore is a comprehensive indicator that integrates ImmuneScore and StromalScore through the ESTIMATE algorithm to quantify the total proportion of immune cells and stromal cells in the tumor microenvironment, and thereby infer the purity of the tumor (the relative proportion of tumor cells in the tissue) [24]. The higher the score, the greater the total proportion of immune cells and stromal cells in the tumor tissue, and the lower the purity of the tumor (the lower the proportion of tumor cells). And a higher ESTIMATEscore indicates an immunotherapy could be less effective [27].

In order to determine whether GPI is related to immune infiltration and the efficacy of immunotherapy, we downloaded the original data from SangerBox and analyzed the immune scores. Then we observed that GPI showed a significant and negative correlation with ImmuneScore in 26 types of cancer (Fig. S2A): LGG, UCEC, CESC, LUAD, ESCA, STES, SARC, KIRP, KIPAN, PRAD, STAD, HNSC, KIRC, LUSC, WT, SKCM-P, SKCM, BLCA, SKCM-M, THCA, PAAD, TGCT, UCS, ACC, KICH, and CHOL(Fig. S2B and Fig. S3A). This indicated that the high expression of GPI is associated with a decrease in the infiltration of immune cells in the tumor microenvironment. And we also found that GPI was significant correlation with StromalScore in 26 types of cancer (Fig. S2D). There are 4 significant positive correlations, such as GBMLGG, LGG, UVM, and LAML(Fig. S2E), suggesting that patients with higher expression levels of GPI may be more resistant to the treatment of these cancers; and there are 22 significant negative correlations, such as UCEC, BRCA, CESC, LUAD, ESCA, STES, SARC, COAD, COADREAD, PRAD, STAD, KIRC, LUSC, THYM, SKCM-P, SKCM, BLCA, SKCM-M, THCA, UCS, ACC, and KICH(Fig. S3B), suggesting that patients with higher expression levels of GPI may respond better to certain treatments and have a better prognosis in these cancers. Furthermore, GPI showed a signiﬁcant correlation with ESTIMATEscore in 32 types of cancer (Fig. S2G). There are 3 significant positive correlations, such as GBMLGG, UVM, and LAML(Fig. S2H), demonstrating that GPI may promote cell differentiation and the formation of a complex tumor microenvironment;while there are 29 significant negative correlations, such as UCEC, BRCA, CESC, LUAD, ESCA, STES, SARC, KIRP, KIPAN, COAD, COADREAD, PRAD, STAD, HNSC, KIRC, LUSC, WT, SKCM-P, SKCM, BLCA, SKCM-M, THCA, MESO, PAAD, TGCT, UCS, ACC, KICH, and CHOL(Fig. S3C), demonstrating that GPI may be involved in the process of forming a less dense tumor microenvironment. GPI may be a predictive indicator for determining the response of tumors to various treatments.

**Fig. 6:**
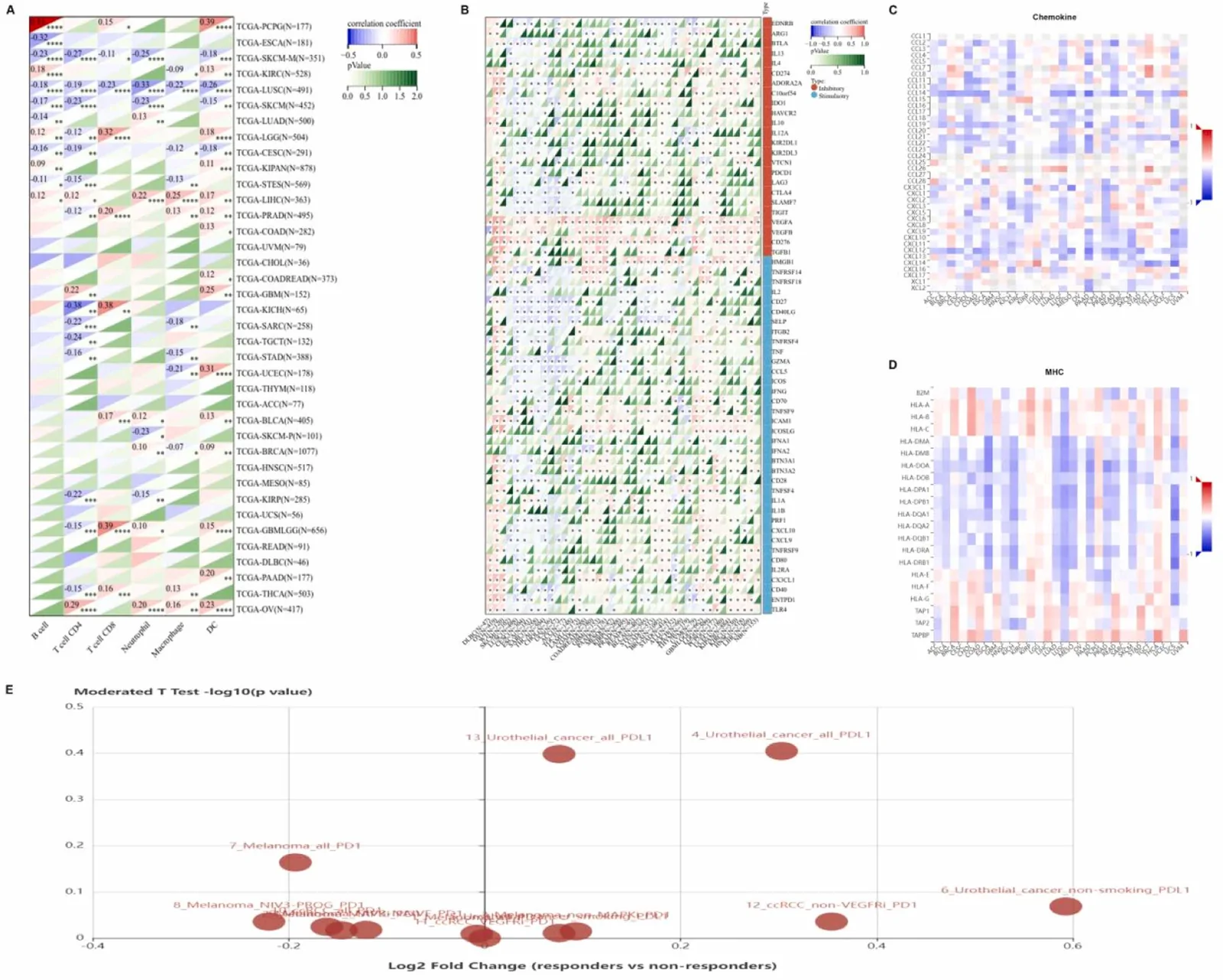
Correlation analysis of GPI with immune infiltration and checkpoint blockade proteins in various types of cancer. (A) The heatmap generated by SangerBox shows the correlation coefficient between the expression of GPI and the infiltration levels of various immune cell types (including B cells, T cells (CD4 and CD8), neutrophils, macrophages, and dendritic cells (DC)) in different cancer types. Positive correlations are indicated by red, and negative correlations by blue. The darkness of the color is proportional to the strength of the correlation. Significant correlations (p < 0.05) are marked with asterisks, and dark green indicates no significant correlation.(B) The heatmap generated by SangerBox illustrates the correlation between the expression of GPI and various genes related to immune checkpoint blockade. The color gradient section represents the correlation coefficient. Red indicates a positive correlation, while blue indicates a negative correlation. Significant correlations are marked with asterisks (*), and dark green indicates no significant correlation. (C) Heatmap of correlations between GPI and chemokine genes.Red indicates a positive correlation, while blue indicates a negative correlation. (D) Heatmap of correlations between GPI and MHC genes.Red indicates a positive correlation, while blue indicates a negative correlation.

Next, we analyzed the correlation between GPI and immune cell inﬁltration in the TME, including B cells, CD4 + T cells, CD8 + T cells, neutrophils, macrophages, and dendritic cells. According to Fig. 6A, GPI was significantly and positively correlated with immune cell infiltration in PCPG, KIRC, LGG, LIHC, PRAD, GBM, UCEC, BLCA, BRCA, GBMLGG, PAAD, THCA, and OV, suggesting that GPI may be useful to promote an efficient immune response against tumors, and those tumors with higher levels of GPI expression may respond better to immunotherapy in these cancers. In contrast, GPI was significantly and negatively correlated with immune cells infiltration in ESCA, SKCM-M, LUSC, SKCM, CESC, STES, SARC, TGCT, STAD, SKCM-P, and KIRP, suggesting that GPI may lead to immunosuppression and tumor escape, and tumors with high GPI expression may be more resistant to immunotherapy in these cancers.

Above all, these results suggest that GPI may be associated with a more active immune response, and it may bring better therapeutic effects in those cancers with a positive correlation. This makes GPI a potential marker for immunogenic tumors, which may respond well to immunotherapy. In contrast, GPI may be involved in immune escape and tumor progression in those cancers with a negative correlation, making GPI a potential target for enhancing immune infiltration and improving cancer treatment outcomes.

The above results indicated that the expression of GPI was associated with immunotherapy. Therefore, we analyzed the correlation between GPI and tumor mutation burden (TMB), microsatellite instability (MSI), or neoantigen load (NEO) to further clarify whether GPI is related to the generation of new antigens. The more genetic mutations (high TMB) there are, or the more types of mutations that can generate new antigens (such as missense mutations related to MSI), the greater the number and diversity of neoantigens [28]. These neoantigens are captured by dendritic cells and presented to T cells, activating anti-tumor immune responses. Therefore, tumors with high TMB (especially MSI-H) typically have a richer neoantigen library and have a higher response rate to immune checkpoint inhibitors (ICI).

We analyzed the correlation between GPI and tumor mutation burden (TMB), microsatellite instability (MSI), or neoantigen load (NEO) by using the SangerBox. It showed that GPI has a significant and positive correlation with TMB in CESC, LUAD, COAD, COADREAD, BRCA, STES, STAD, PRAD, and READ(Fig. S2C), indicating that the high expression of GPI was associated with a greater number of mutations in these 9 types of tumors. In contrast, it showed that GPI has a significant and negative correlation with TMB in LUSC(Fig. S2C), indicating that the high expression of GPI is associated with a lesser number of mutations in LUSC. This indicated that GPI might be a potential biomarker for predicting the efficacy of these 10 types of tumor immunotherapies. And GPI showed a significant and positive correlation with MRI in CESC, COADREAD, STES, SARC, STAD, UCEC, TGCT, UVM(Fig. S2F), suggesting that patients with higher expression levels of GPI are more likely to benefit from immunotherapy in these 8 types of tumors. While GPI showed a significant and negative correlation with MRI in GBMLGG, THCA(Fig. S2F), suggesting that patients with higher expression levels of GPI are less likely to benefit from immunotherapy in these 2 types of tumors. Then we found that GPI was significantly and positively correlated with NEO in LUAD, COAD, COADREAD(Fig. S2I), revealing that patients with higher expression levels of GPI can achieve better therapeutic effects in immunotherapy. Whereas, GPI was significantly and negatively correlated with NEO in GBM(Fig. S2I), revealing that patients with higher expression levels of GPI can achieve worse therapeutic effects in immunotherapy.

Next, we analyzed the correlation between GPI and immune checkpoint blocking proteins, as these proteins are the targets of immune checkpoint blocking therapies. And we found GPI showed a significant positive correlation with most of the immune checkpoint proteins in DLBC, OV, WT, COAD, COADREAD, GBM, PAAD, MESO, PRAD, READ, BLCA, STES, LUAD, BRCA, STAD, UVM, GBMLGG, LGG, PCPG, UCEC, KIPAN, KIRC, HNSC, LIHC, and NB(Neuroblastoma)(Fig. 6B). This suggested that GPI may be involved in the immune escape process of tumors, making it an important biomarker for predicting prognosis and immunotherapy. Therefore, patients with higher levels of GPI expression are likely to benefit from immune checkpoint inhibitor therapy in the treatment of these cancers. In contrast, GPI showed a significant negative correlation with most of the immune checkpoint proteins in LUSC, CESC, ESCA, THCA, CHOL, and UCS(Fig. 6B).This suggested that GPI may be linked to a more active immune environment within the TME of these tumors.

The association between GPI and immune-related genes can help us understand the response of tumors with high GPI expression to immunotherapy. Chemokines, through gradient concentration signals, recruit immune cells to specific tissues (such as the tumor microenvironment), directly regulating the tumor immune response and progression. They serve as the key bridge connecting "immunocyte recruitment" and "tumor microenvironment remodeling" [29].The MHC gene is a core gene family encoding the antigen-presenting molecules on the surface of immune cells. It directly determines whether tumor neoantigens (NEO) can be recognized by T cells, and is a crucial hub connecting "tumor mutations" and "immune responses". This is crucial for initiating an immune response against tumor cells [30].As was shown in Fig. 6C, we found that GPI was positively correlated with immune chemokines in BRCA, GBM, LGG, LIHC, OV, STAD, and TGCT, suggesting that high expression of GPI may promote the expression of chemokines. In contrast, GPI was negatively correlated with immune chemokines in BLCA, CHOL, COAD, ESCA, KICH, LUSC, READ, and UCS, suggesting that high expression of GPI may restrain the expression of chemokines. Both of these situations may further affect the recruitment process of immune cells. And through Fig. 6D, we found that GPI was positively correlated with BRCA, CHOL, KIRP, LGG, LIHC, OV, PCPG, SARC, STAD, THCA, UCES and UVM MHC genes. This indicates that the high expression of GPI is associated with the up-regulated expression of MHC genes, which may enhance antigen presentation and immune recognition. Whereas, we found that GPI was negatively correlated with BLCA, CESC, ESCA, HNSC, KICH, LUAD, LUSC, MESO, PAAD, PRAD, SKCM, and UCS MHC genes. This indicates that the high expression of GPI is associated with the down-regulated expression of MHC genes, which may reduce antigen presentation and immune recognition.

Furthermore, in order to determine whether there are differences in the expression of GPI between responders and non-responders to checkpoint blockade (such as anti-PDL1 and anti-PD1), we utilized TISIDB, which is an integrated portal for tumor immune system interaction databases. This database collects transcriptomic and genomic analysis datasets of preprocessed tumor biopsy samples from both responders and non-responders to immunotherapy. We used the TISIDB portal to analyze the corelation between GPI expression and immunotherapy response. Then, we found that immunotherapeutic responders had higher GPI expression than non-responders in Melanoma_non-MAPKi_PD1, Melanoma_NIV3-NAIVE_PD1 (nivolumab),and ccRCC_all_PD1 (nivolumab), suggesting that GPI is up-regulated in these responders and might serve as a prognostic marker in these cancers. In contrast, immunotherapeutic responders had lower GPI expression than non-responders in Melanoma_all_PD1(pembrolizumab and nivolumab), Melanoma_MAPKi_PD1 (pembrolizumab and nivolumab), Urothelial_cancer_all_PDL1 (atezolizumab), Urothelial_cancer_smoking_PDL1(atezolizumab), Urothelial_cancer_non-smoking_PDL1 (atezolizumab), Melanoma_all_PD1 (nivolumab), Melanoma_NIV3-PROG_PD1 (nivolumab),ccRCC_non-VEGFRi_PD1(nivolumab), and Urothelial_cancer_all_PDL1(atezolizumab), indicating that GPI is down-regulated in these responders and may be related to the resistance to immunotherapy (Fig. 6E and Table 1). Notably, no comparison achieved statistical significance (all raw P > 0.05 after FDR multiple testing correction). The observed directional fold changes were only exploratory trends without statistical reliability. These non-significant trends cannot support the claim that GPI expression correlates with immunotherapy response or mediates treatment resistance.Thus, further validations are required to establish the definite relationship between GPI expression, immunotherapy response and treatment resistance.

**Table 1:** The difference in GPI between immunotherapeutic responders and non-responders.

**GPI**	**No**	**PMID**	**Cancer type**	**Group**	**Drug**	**#Responders**	**#Non-responders**	**Log2(Fold Change)**	**Pvalue**
Up-regulated	3	26997480	Melanoma	non-MAPKi	Anti-PD-1(pembrolizumab and nivolumab)	8	7	0.093	0.967
	4	28552987	Urothelial cancer	all	Anti-PD-L1(atezolizumab)	9	16	0.303	0.394
	5	28552987	Urothelial cancer	smoking	Anti-PD-L1(atezolizumab)	5	9	0.076	0.975
	6	28552987	Urothelial cancer	non-smoking	Anti-PD-L1(atezolizumab)	4	7	0.593	0.854
	12	29301960	Clear cell renal cellcarcinoma (ccRCC)	non-VEGFRi	Anti-PD-1(nivolumab)	2	8	0.354	0.921
	13	29443960	Urothelial cancer	all	Anti-PD-L1(atezolizumab)	68	230	0.076	0.4
_Non-regulated_	11	29301960	Clear cell renal cell carcinoma (ccRCC)	VEGFRi	Anti-PD-1(nivolumab)	2	0	0	1
Down-regulated	1	26997480	Melanoma	all	Anti-PD-1(pembrolizumab	14	12	-0.008	0.979
	2	26997480	Melanoma	MAPKi	and nivolumab) Anti-PD-1(pembrolizumab and nivolumab)	6	5	-0.146	0.962
	7	29033130	Melanoma	all	Anti-PD-1(nivolumab)	26	23	-0.193	0.686
	8	29033130	Melanoma	NIV3-PROG	Anti-PD-1(nivolumab)	15	11	-0.22	0.921
	9	29033130	Melanoma	NIV3-NAIVE	Anti-PD-1(nivolumab)	11	12	-0.121	0.96
	10	29301960	Clear cell renal cellcarcinoma (ccRCC)	all	Anti-PD-1(nivolumab)	4	8	-0.161	0.945

Table 1 The difference in GPI between immunotherapeutic responders and non-responders.All inter-group comparisons adopted moderated *t*-test; no cohort reached raw P < 0.05 or FDR-q < 0.05. Sample size of each subgroup is listed in the table. All fold-change differences are exploratory unvalidated trends without statistical significance.

Overall, these results highlight the potential significance of GPI in cancer development and treatment. Given its association with immune cell infiltration and immune treatment response, GPI can be explored as a potential therapeutic target and/or a biomarker for predicting the treatment efficacy of cancer patients.

### GPI serves as a predictive biomarker for cancer treatment responses and a screening indicator for potential targeted drugs

**Fig. 7:**
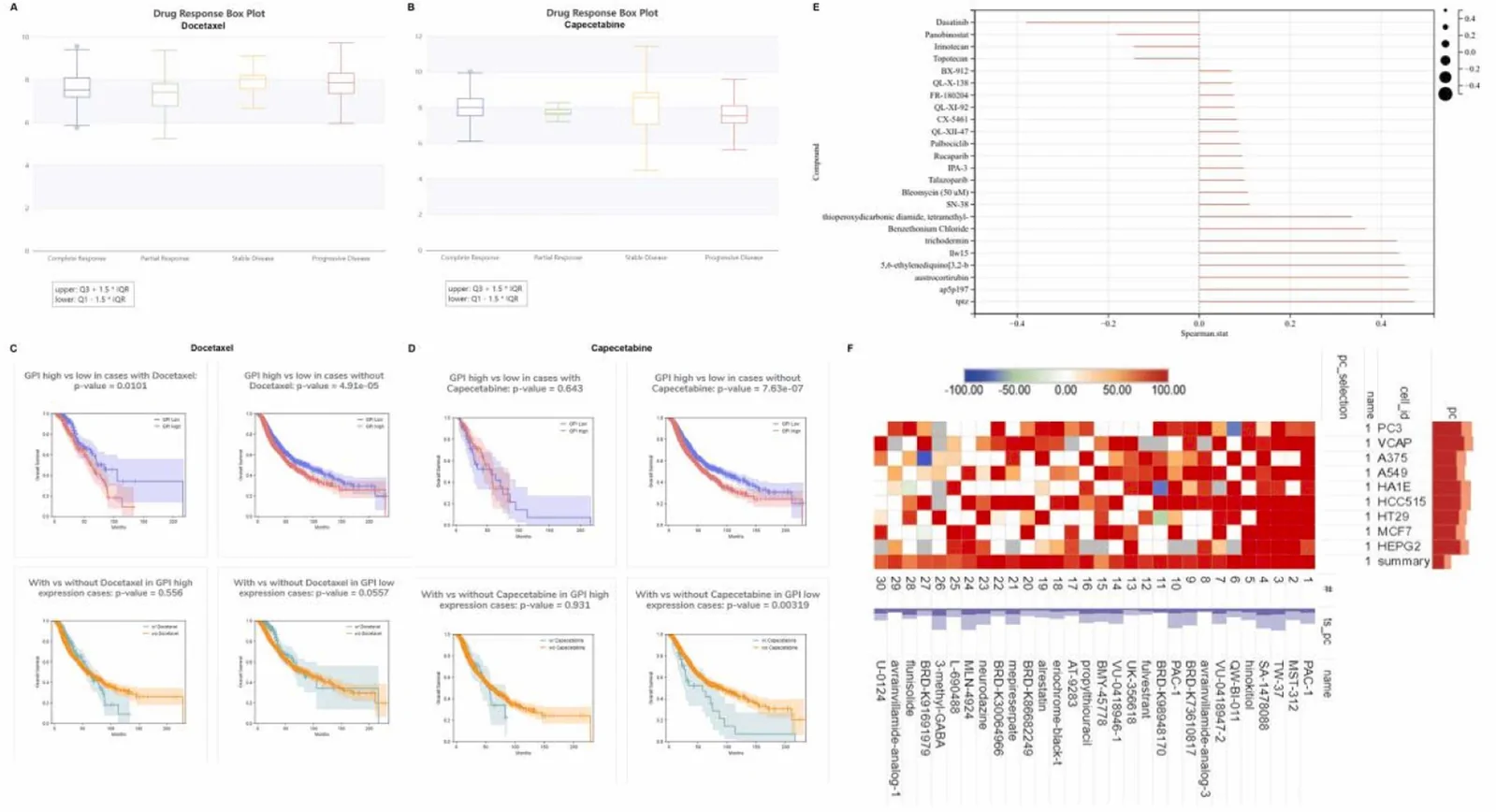
Correlation analysis of GPI with treatment, as well as compound research. (A-B) The TCGA drug analysis of GPI in various types of cancers with docetaxel(A) and capecetabine(B).The horizontal axis in the figure represents the four levels of tumor's response to the drug treatment, and the vertical axis shows the expression level of the GPI gene.(C-D)Correlation analysis between the expression level of GPI and the overall survival period of patients treated with docetaxel(C) and capecitabine(D)(95% Confidence Interval).(E)Bar charts showing Spearman correlations between GPI grouping and mRNA changes induced by RNAactDrug drugs.(F) A heat map shows the top 30 compounds. The experimental-induced transcriptional changes of these compounds are opposite to the changes influenced by the medium GPI expression group. Color bars and block colors indicate similarity scores.

In order to investigate whether GPI can serve as a predictive marker for the efficacy of certain anti-tumor drugs, we conducted TCGA drug analysis using GEPIA3.We obtained the differences in GPI expression among patients with different drug reactions. And there are two drugs that have a significant correlation with the expression of GPI(p value<0.05):docetaxel and capecetabine.As shown in Fig. 7A, the median expression level of GPI in the stability disease group was the highest, suggesting that high expression of GPI might be associated with "disease non-response" after docetaxel treatment. And the GPI expression in the complete/ partial response group was relatively lower, indicating that tumors with low GPI expression are more sensitive to docetaxel. Tumors with low expression of GPI are more sensitive to docetaxel (with higher rates of complete / partial remission), while tumors with high expression of GPI are more likely to have stable / progressive disease, suggesting that GPI can serve as a preliminary exploratory signal linked to treatment-associated outcomes for the efficacy of docetaxel. According to Fig. 7B, the median expression level of GPI in the stability disease group was the highest and showed significant fluctuations, suggesting that high expression of GPI might be associated with the "unresolved disease" after capecitabine treatment. While the GPI expression in the partial response group was the lowest and most concentrated, suggesting that tumors with low GPI expression are more sensitive to capecitabine. Therefore, tumors with low expression of GPI are more sensitive to capecitabine and docetaxel(with higher rates of complete / partial remission), while tumors with high expression of GPI are more likely to have stable/ progressive disease, suggesting that GPI can serve as a preliminary exploratory signal linked to treatment-associated outcomes for the efficacy of capecitabine and docetaxel. Stratified analyses based on treatment status revealed preliminary associations between GPI expression and clinical outcomes. However, these exploratory trends cannot confirm a definite predictive or independent prognostic value of GPI, due to the lack of formal treatment-GPI interaction analysis and insufficient adjustment for clinical confounding factors, including cancer stage and treatment indication. Further prospective validation and rigorous multivariate modeling are required to verify its clinical utility.

Next,we conducted an analysis to investigate the correlation between the expression level of GPI and the overall survival period of patients treated with docetaxel and capecitabine. Fig. 7C showed that the survival rate of the group with low expression of GPI was significantly higher than that of the group with high expression (P = 0.0101 < 0.05) among the patients treated with docetaxel, suggesting that patients with low GPI expression have a better survival prognosis when treated with docetaxel. For patients who did not receive docetaxel treatment, the survival rate of the low-expression group of GPI was significantly higher than that of the high-expression group (P = 4.91e-05 < 0.001), indicating that regardless of whether docetaxel was used or not, high expression of GPI is an adverse prognostic factor for cancer patients.For patients with high expression of GPI, there was no significant difference in survival rates between the two groups (P = 0.556 > 0.05), suggesting that the use of docetaxel does not improve survival in patients with high GPI expression.In patients with low expression of GPI, the survival rates of the two groups were close to the critical significance (P = 0.0557 ≈ 0.05), suggesting that might improve survival in patients with low GPI expression. Through Kaplan-Meier survival analysis, it was clarified that the expression of GPI has a dual value in terms of docetaxel treatment and tumor prognosis: GPI is a potential adverse prognostic biomarker, and GPI is a predictive marker for the efficacy of docetaxel treatment. The results support incorporating GPI expression testing into the medication decision-making process for docetaxel: for patients with low GPI expression, docetaxel is recommended to potentially improve survival; for patients with high GPI expression, it is suggested to avoid docetaxel and prefer alternative treatment options. From Fig. 7D, we found that among the patients treated with capecitabine, there was no significant difference in survival rates between the two groups (P = 0.643 > 0.05), suggesting that the level of GPI expression does not affect the survival prognosis when receiving capecitabine treatment. Among the patients who did not receive capecitabine treatment, the survival rate of the low-expression group of GPI was significantly higher than that of the high-expression group (P = 7.63e-07 < 0.001), indicating that high expression of GPI is an adverse prognostic factor for tumor patients (independent of whether capecitabine was used or not).For patients with high expression of GPI, there was no significant difference in survival rates between the "using capecitabine" group and the "not using capecitabine" group (P = 0.931 > 0.05), suggesting that GPI high expression in patients cannot be improved by using capecitabine in terms of survival. For patients with low expression of GPI, the survival rate of the group using capecitabine was significantly higher than that of the non-users (P = 0.00319 < 0.01), indicating that GPI low-expression patients can significantly improve survival when using capecitabine. The Kaplan-Meier survival analysis results indicate that GPI is a precise predictor of the efficacy of capecitabine. In tumor treatment, the precise use of capecitabine can be achieved by detecting the expression level of GPI in tumor tissues: for patients with low GPI expression, capecitabine can be used as the preferred treatment option to prolong the overall survival period; for patients with high GPI expression, capecitabine should be avoided and alternative treatment options should be selected instead.

Furthermore, the relationship between GPI and drug sensitivity was conﬁrmed using RNAactDrug. The Spearman correlation coefficient of thioperyldicarbonic diamide and Benzethonium Chloride is closer to −0.4(Fig. 7E), indicating that the transcriptional changes of these compounds have a stronger negative correlation with the high expression feature of GPI, and have a greater potential to reverse the high expression of GPI, thereby being able to improve the therapeutic effect of GPI-overexpressing tumors.

Using the cMap tool, we identified 30 compounds that could cause transcriptional changes opposite to the increase in GPI expression in 9 different tumor cell lines(Fig. 7F). These compounds were ultimately determined to have potential for targeting GPI.

### GPI exerts a pro-oncogenic effect in tumor cells

**Fig. 8:**
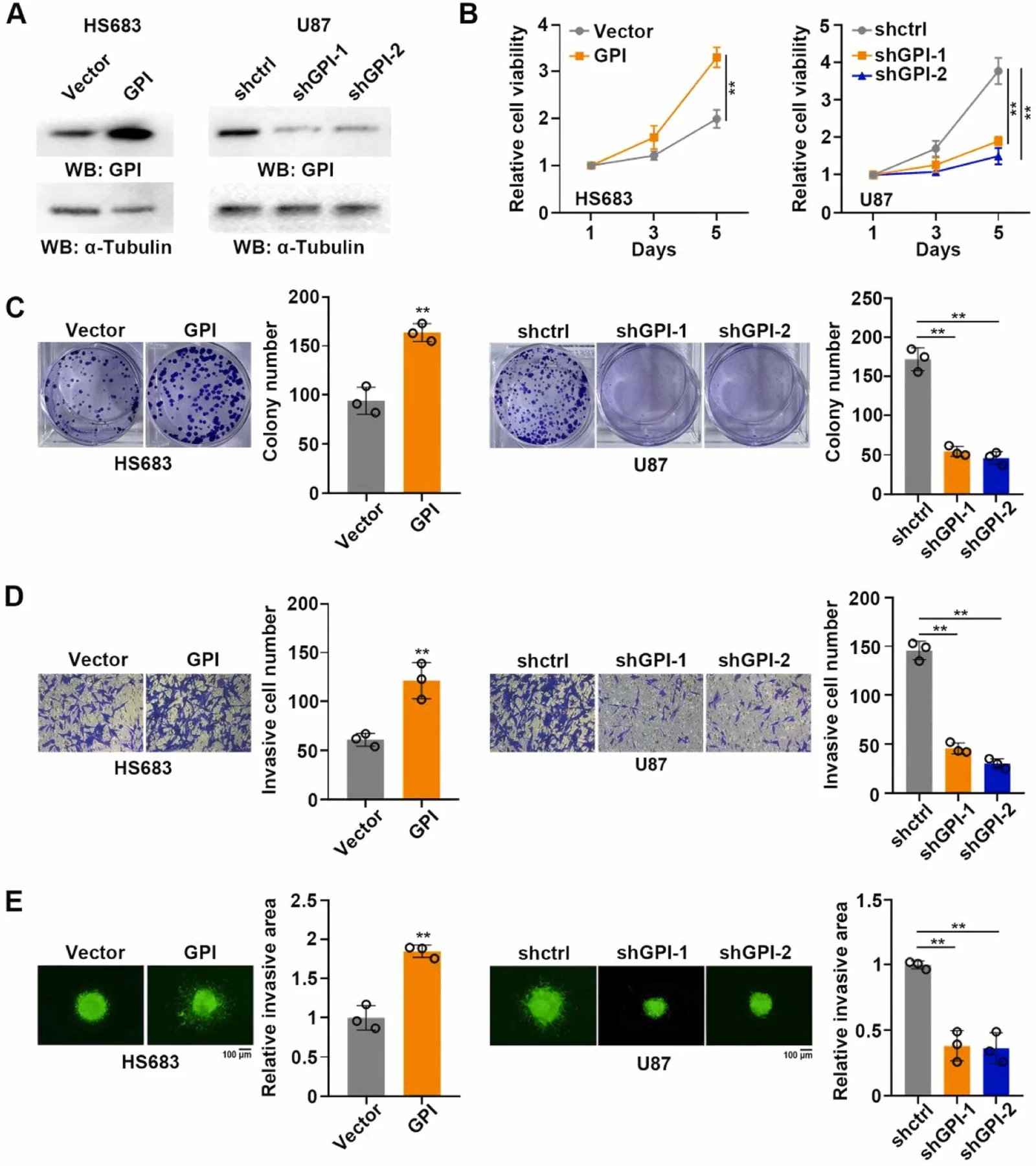
The oncogenic effect of GPI in tumor cells. (A) Expression verification of GPI (Western Blot).Overexpression of GPI in HS683 cells (left), knockdown of GPI in U87 cells (right), with α-Tubulin used as the internal control.(B). Cell proliferation ability experiments of HS683(left) cells and U87(right) cells.(C)Clonogenicity and proliferative capacity experiments of HS683(left) cells and U87(right).(D) Cell invasion ability experiments of HS683(left) cells and U87(right) cells.(E) Analysis of the invasive regions of HS683(left) cells and U87(right) cells (fluorescence experiment).All data represent mean ±SD from 3 independent biological replicates;*p < 0.05, **p < 0.01 (Student’s *t*-test). Scale bar = 100 µm for fluorescence invasion images (Panel E). Uncropped Western blots are supplied in Supplementary WB.

Through the above-mentioned relevant analysis, we found that GPI played a crucial role in the occurrence, development, treatment and prognosis of tumors. To further validate the above analysis, we conducted a cell culture experiment on human LGG and GBM. GPI was overexpressed in LGG cell line (HS683), and depleted in GBM cell line (U87) (Fig. 8A). Then, we detected the cell proliferation ability using the CCK-8 method. In the HS683 cells, the absorbance value of the GPI group on the 5th day of culture was significantly higher than that of the Vector group; and in the U87 cells, the absorbance values of the shGPI-1 and shGPI-2 groups on the 5th day were significantly lower than those of the shctrl group(Fig. 8B), suggesting that GPI can promote tumor cell proliferation.

Next, in the cloning formation experiment, the number of clones in the GPI group of HS683 cells was significantly higher than that in the Vector group. While in U87 cells, the number of clones in the shGPI-1 and shGPI-2 groups was significantly lower than that in the shctrl group(Fig. 8C). These results indicated that GPI can enhance the clonogenic ability of tumor cells, that is, it can improve their anchorage-independent growth ability.

Finally, the cell invasion ability was verified through the Transwell assay and the analysis of the fluorescent invasion area. In the Transwell assay, the number of invasive cells in the GPI group of HS683 cells was significantly higher than that in the Vector group, and the number of invasive cells in the shGPI-1 and shGPI-2 groups of U87 cells was significantly lower than that in the shctrl group(Fig. 8D). The results of the fluorescence invasion area analysis were consistent with this: the invasion area of HS683 cells in the GPI group was significantly larger than that in the Vector group; the invasion area of U87 cells in the shGPI-1 and shGPI-2 groups was significantly smaller than that in the shctrl group(Fig. 8E). These results confirmed that GPI could significantly enhance the invasive ability of tumor cells.

Above all, GPI plays a role in promoting cancer in tumor cells: Overexpression of GPI will enhance cell proliferation, clonal formation, and invasive ability.

## Discussion

GPI expression was significantly dysregulated in most cancer types, with over-expression in 29 tumors (e.g., LUAD, BRCA, LUSC) and down-regulation in 3 (LGG, THCA, WT). This dual expression pattern suggests GPI may exert cancer-type-specific roles—oncogenic in most cancers and tumor-suppressive in a subset. Notably, GPI overexpression correlated with advanced tumor stages (T, N, M stages) and poor prognosis in LUAD, LGG, SKCM, and other cancers, consistent with previous reports in breast and lung cancer. For example, GPI expression increased from M0 to M1 in LUAD, indicating its involvement in distant metastasis. These results support GPI as a potential diagnostic biomarker for distinguishing tumor from normal tissues and a prognostic indicator for risk stratification [3].

Gender differences in GPI expression were observed in KIRP, KIPAN, KIRC, LIHC, and LUSC, highlighting the need to consider gender when using GPI as a biomarker. This may be attributed to gender-specific metabolic differences or hormone regulation, which warrants further investigation [31].

GPI genetic alterations were common across cancers, with missense mutations as the main type and UCEC showing the highest mutation rate. Hotspot mutations (K211E/M/N, A540T) in the PGI domain suggest these sites may be critical for GPI’s oncogenic function [32]. Copy number gains were the predominant genomic change, positively regulating GPI mRNA expression in 20 cancers (e.g., LUAD, BRCA, ESCA), indicating that genomic amplification is a key mechanism driving GPI overexpression. Additionally, GPI mRNA expression was negatively correlated with DNA methylation in UCEC, STAD, LUSC, and other cancers, suggesting epigenetic silencing may contribute to GPI down-regulation in certain tumors. These findings imply GPI dysregulation is driven by both genetic and epigenetic mechanisms, providing potential targets for therapeutic intervention [33].

GPI was positively correlated with multiple stemness indices (RNAss, DNAss, EREG-METHss) in most cancers, indicating its association with cancer stem cell (CSC) characteristics. CSCs are responsible for tumor initiation, metastasis, and drug resistance [34], so GPI may promote tumor aggressiveness by enhancing stemness. This is supported by GPI’s co-expressed genes being enriched in metabolic pathways (glycolysis, oxidative phosphorylation, mitochondrial electron transport) and mitochondrial functions. The top interacting protein, TPI1, a glycolytic enzyme, showed moderate positive correlation with GPI, suggesting GPI may cooperate with glycolytic pathways to support tumor metabolic reprogramming—an essential process for CSC survival and proliferation [35].

GPI showed significant correlations with immune/stromal/ESTIMATE scores in most cancers. Notably, GPI overexpression was associated with reduced immune scores in 26 cancers, suggesting it may suppress immune cell infiltration and promote immunosuppression. It consistents with reports that metabolic enzymes can modulate the immune microenvironment [36], [37].This is consistent with GPI’s negative correlation with CD4 + T cells, CD8 + T cells, and dendritic cells in ESCA, LUSC, and CESC, which may facilitate tumor immune escape [38]. Conversely, GPI was positively correlated with immune cell infiltration in PCPG, KIRC, and LGG, indicating cancer-type-specific immune regulatory roles.

GPI’s association with TMB, MSI, and immune checkpoint proteins (e.g., PD-L1) further supports its role in immunotherapy response. High GPI expression correlated with high TMB in LUAD, COAD, and STAD, predicting better immunotherapy efficacy, while the opposite was observed in LUSC. Additionally, GPI expression differed between immunotherapy responders and non-responders in melanoma and renal cell carcinoma. Responders had higher GPI expression in Melanoma_non-MAPKi_PD1 and ccRCC_all_PD1, but lower expression in Melanoma_all_PD1 and Urothelial_cancer_all_PDL1. This discrepancy may reflect cancer-type-specific immune landscapes or treatment regimens (e.g., combination with MAPK inhibitors) [39], emphasizing the need for cancer-specific GPI-based immunotherapy stratification, suggesting it may serve as an immunotherapy predictive biomarker. Non-statistically significant exploratory trends implied potential links between GPI expression and immunotherapy response, which requires prospective clinical cohort validatio. These findings highlight GPI’s potential to guide immunotherapy selection.

GPI was identified as a predictive marker for docetaxel and capecitabine efficacy. Low GPI expression correlated with better response to these chemotherapeutic agents and longer survival, while high GPI expression predicted resistance. Docetaxel inhibits microtubule depolymerization[40], and GPI overexpression may promote drug efflux or enhance cell survival signaling—similar to multidrug resistance protein 1 (MDR1)-mediated chemotherapy resistance [41].Capecitabine is a prodrug of 5-fluorouracil (5-FU), which targets thymidylate synthase [42]; GPI may regulate pyrimidine metabolism to counteract 5-FU’s effects, though this requires further validation.

RNAactDrug analysis identified thioperoxydicarbonic diamide and Benzethonium Chloride as potential compounds to reverse GPI overexpression, providing candidate drugs for GPI-high tumors. In vitro experiments confirmed GPI overexpression enhanced LGG cell proliferation, clonogenicity, and invasion, while knockdown reversed these effects—validating GPI’s pro-oncogenic function and supporting its potential as a therapeutic target (e.g., via siRNA-mediated knockdown or small-molecule inhibitors) [43].

This study has limitations. First, bioinformatics analysis relies on public databases, and potential biases in data collection and processing should be considered [44]. Second, in vitro experiments were limited to LGG and GBM cell lines; validation in other cancer types and in vivo models is needed [45], [46].Third, the molecular mechanisms underlying GPI’s regulation of stemness, immune microenvironment, and therapeutic resistance require further exploration. Future studies should focus on: (1) validating GPI’s biomarker role in clinical samples; (2) investigating the molecular pathways mediating GPI’s oncogenic effects(e.g., PI3K/Akt or MAPK signaling) [47]; (3) developing GPI-targeted therapies and combining them with chemotherapy or immunotherapy [48];(4) exploring gender-specific regulatory mechanisms of GPI.

In conclusion, this study demonstrates that GPI is a key regulator of pan-cancer progression, with dysregulated expression, genetic/epigenetic alterations, and associations with stemness, immune microenvironment, and therapeutic response. GPI holds promise as a diagnostic, prognostic, and therapeutic biomarker, providing new insights for personalized cancer treatment.

## Conclusions

Glucose-6-phosphate isomerase (GPI), a key metabolic enzyme, displays dysregulated expression in the majority of cancer types, being upregulated in 29 tumor types (e.g., LUAD, BRCA, LUSC) and downregulated in 3 (e.g., LGG, THCA). High GPI expression correlates with advanced tumor stages, metastasis and poor clinical prognosis in cancers including LUAD, LGG and SKCM. Genetic alterations (e.g., missense mutations, copy number gains) and epigenetic regulation (e.g., DNA methylation) jointly drive such dysregulation of GPI, which is also positively associated with cancer stemness indices (RNAss, DNAss) and RNA modifications (m1A, m5C, m6A) across multiple cancer types. Its co-expressed genes are enriched in core metabolic pathways such as glycolysis and oxidative phosphorylation, with TPI1 identified as its top interacting protein. GPI modulates the tumor immune microenvironment by correlating with immune, stromal and ESTIMATE scores as well as immune cell infiltration; it is also linked to immune checkpoint proteins, TMB, MSI and immunotherapy response, exhibiting cancer-type-specific immune regulatory roles. Furthermore, GPI functions as a potential prognostic biomarker for the efficacy of docetaxel and capecitabine, with low GPI expression indicative of a better therapeutic response, and several potential GPI-targeting compounds (e.g., thioperyldicarbonic diamide) have been screened out in this study. In vitro experiments validated that GPI overexpression significantly enhances the proliferation, clonogenicity and invasive capacity of LGG cells (HS683) and GBM cells (U87), while GPI knockdown reverses these malignant phenotypes. Collectively, GPI acts as a pivotal pan-cancer pro-oncogenic factor and holds great promise as a multi-functional biomarker for cancer diagnosis, prognosis assessment and therapeutic response prediction, thereby providing novel insights for the development of personalized cancer treatment strategies.

## CRediT authorship contribution statement

W.H.: Bioinformatics analysis, Writing – original draft. Y.G.: Bioinformatics analysis, Writing – original draft. Z.L.: Cell culture experiments, Writing – original draft, Funding acquisition. Z.D.: Data curation, Formal analysis, Visualization. J.T.: Data curation, Formal analysis, Visualization. A.A. Politis: Data curation, Formal analysis, Visualization. F.V. Grilli: Data curation, Formal analysis, Visualization. A. Di Stefano: Data curation, Formal analysis, Visualization. X.W.: Conceptualization, Methodology, Supervision, Writing – review & editing, Funding acquisition. J.Z.: Conceptualization, Methodology, Supervision, Writing – review & editing, Funding acquisition. J.Y.: Conceptualization, Methodology, Supervision, Writing – review & editing, Funding acquisition. All authors: Writing – review & editing, Validation.

## Funding

This research work was supported by: 10.13039/501100001809National Natural Science Foundation of China (82303874); Suqian Sci&Tech Program (No. KY202402).

## Declaration of Competing Interest

The authors declare that they have no known competing financial interests or personal relationships that could have appeared to influence the work reported in this paper.

## Data Availability

All raw pan-cancer transcriptome, genomic and clinical data analyzed in this study are publicly available via open-access databases: GTEx v8 (https://gtexportal.org/), TCGA Firehose Legacy (https://xena.ucsc.edu/), cBioPortal (https://www.cbioportal.org/), TIMER2.0 (http://timer.cistrome.org/), GEPIA2/3 (http://gepia.cancer-pku.cn/), TISIDB (http://cis.hku.hk/TISIDB/), RNAactDrug (http://bio-bigdata.hrbmu.edu.cn/RNAactDrug/). All R analysis scripts, normalized gene expression matrices and figure parameter configuration files are provided as supplementary files. No raw sequencing data were generated in this study.
