# Supplementary material for: GPI: A pan-cancer biomarker integrating genetic alterations, immune infiltration, and therapeutic response prediction
Source: Glob Med Genet. 2026 Sep 10;13(3):100127. doi: 10.1016/j.gmg.2026.100127 (PMC13598195; doi:10.1016/j.gmg.2026.100127)
Supplement: Supplementary file 1 [file mmc1.docx]

Figure S1	GPI acts as a core hub in the glycolysis and pentose phosphate pathway (PPP) co-expression network.(A) Protein-protein interaction (PPI) network of key glycolytic and PPP genes, including GPI, TPI1, TKT, G6PD, TKTL2, PFKL, PFKM, PFKP, MPI, TALDO1, and H6PD.(B) Scatter plots showing the correlation between GPI and indicated genes (MPI, TKT, TPI1, G6PD, H6PD) based on log2(TPM) expression values. Pearson correlation coefficient (R) and P-value are shown for each comparison.

Figure S2	Correlation analysis of GPI with immune infiltration in various types of cancer. (A) The radar chart illustrates the correlation between the expression of GPI and ImmuneScore. The analysis was conducted using SangerBox. The figure shows the correlation coefficients, and different cancer types are annotated around it. (B) These scatter plot combined with marginal distribution histograms are used to demonstrate the correlation between the expression level of the GPI gene and the ImmuneScore(Signiﬁcant correlations (p < 0.05)) in LGG, UCEC, LUAD, and ESCA. (C) The bar chart illustrates the correlation between the expression of GPI and tumor mutation burden (TMB). The bar colors represent the pValue. The redder the color, the more significant the correlation and the more reliable the result. (D)The radar chart illustrates the correlation between the expression of GPI and StromalScore.(E)These scatter plot combined with marginal distribution histograms are used to demonstrate the correlation between the expression level of the GPI gene and the StromalScore(Signiﬁcant positive (p < 0.05)). (F) The bar chart illustrates the correlation between the expression of GPI and microsatellite instability (MSI) across various cancers. The bar colors represent the pValue. The redder the color, the more significant the correlation and the more reliable the result.(G)The radar chart illustrates the correlation between the expression of GPI and ESTIMATEScore.(H)These scatter plot combined with marginal distribution histograms are used to demonstrate the correlation between the expression level of the GPI gene and the ESTIMATEScore(Signiﬁcant positive (p < 0.05)).(I) The bar chart illustrates the correlation between the expression of GPI and neoantigen burden (NEO) across various cancers. The bar colors represent the pValue. The redder the color, the more significant the correlation and the more reliable the result.

Figure S3	Correlation analysis of GPI with immune infiltration in various types of cancer. (A)These scatter plot combined with marginal distribution histograms are used to demonstrate the correlation between the expression level of the GPI gene and the ImmuneScore(Signiﬁcant correlations (p < 0.05)).(B)These scatter plot combined with marginal distribution histograms are used to demonstrate the correlation between the expression level of the GPI gene and the StromalScore(Signiﬁcant negative (p < 0.05)).(C)These scatter plot combined with marginal distribution histograms are used to demonstrate the correlation between the expression level of the GPI gene and the ESTIMATEScore(Signiﬁcant negative (p < 0.05)).
